# Development and Characterisation of Pasta Enriched with Carrot Powder

**DOI:** 10.3390/foods15020289

**Published:** 2026-01-13

**Authors:** Sofia G. Florença, Ana C. Ferrão, Filipa P. Costa, Raquel P. F. Guiné

**Affiliations:** CERNAS-IPV Research Centre, Polytechnic University of Viseu, Repeses, 3504-510 Viseu, Portugal; sofiaflorenca@outlook.com (S.G.F.); aferrao@esav.ipv.pt (A.C.F.); filipa.pereira@esav.ipv.pt (F.P.C.)

**Keywords:** new food, gluten-free, dried carrot, cooking loss, texture, sensorial analysis

## Abstract

Pasta is a staple food and is a typical commodity worldwide. However, some people with celiac disease or gluten intolerance cannot consume pasta formulated with wheat flour. This work aimed to develop and characterise pasta samples made from wheat and buckwheat flours fortified with carrot powder at concentrations of 5% and 10%. The developed pasta samples were analysed for drying and hydration characteristics, for cooking properties, pasting properties, colour, texture, and sensory attributes. The results showed that the wheat-based pastas had better hydration and cooking properties, and that the gluten-free pastas were less cohesive. Concerning hardness, the addition of carrot powder produced opposite results for the wheat- and the buckwheat-based pastas. The gluten-free samples had higher pasting temperatures and peak viscosities and were also darker; however, lightness, redness, and yellowness increased with the addition of carrot powder. The gluten-free pastas were richer in terms of nutrients, carotenoids, and phenolic compounds due to the presence of buckwheat instead of wheat flour, and the increased addition of carrot powder also contributed to the increase in these nutrients. The sensory evaluation revealed that judges preferred the wheat-based pasta samples over the buckwheat counterparts, and the addition of carrot powder at the highest percentage significantly improved the sensorial assessment. In conclusion, the pasta samples formulated have high nutritional importance, and sensorial acceptance was increased with the addition of carrot powder.

## 1. Introduction

Pasta is a staple food in many parts of the globe and across various civilisations, consumed by people from diverse sociodemographic and economic backgrounds. Consumer acceptance of a high-quality pasta is intimately associated with its physical as well as sensorial characteristics. Nevertheless, an increasing number of consumers attribute added value to food products, particularly pasta, based on their nutritional value, health benefits, or traceability [1]. Important pasta characteristics include colour, texture, cooking characteristics, flavour, mouthfeel, nutritional value, and presence of bioactive components with health-enhancing properties [2].

Diverse ingredients can be incorporated into pasta, serving various objectives, such as enriching it with proteins, fibres, or antioxidants, and other valuable components [3,4,5,6,7]. It is also possible to enhance the nutritional and health-promoting properties of pasta through the addition of valuable compounds obtained through the processing of food industry by-products [8,9,10,11]. The valorisation of food waste and incorporation of residues into new food products through an upcycling strategy offers several advantages, particularly in terms of environmental, social, and economic benefits. Reducing food waste and feeding the global population are strategic areas compiled in the Sustainable Development Goals of the United Nations [12]. Many other ingredients have been added to pasta to improve its nutritional and functional properties, thereby enhancing its bioactivity. Ingredients typically containing phenolic compounds or other antioxidant components have been successfully added to pasta, like, for example, some fruits or vegetables rich in antioxidants [13,14,15,16,17]. Also, some advocate that the addition of vegetables to pasta formulations can contribute to vegetable intake [16]. Carrots are a widely available vegetable rich in carotenoids and phenolic compounds, which possess antioxidant activity [18,19,20]. They have been used in the form of powder to enrich certain food products, such as cheese [18], cake [21], or ice cream cones [22].

Pasta is a widely consumed food commodity, but its traditional form is not suitable for consumption by people with celiac disease or those with gluten intolerance, as pasta made with wheat flour contains gluten. Celiac disease encompasses an immune-mediated damage in the gut mucosa caused by the presence of gluten in the foods ingested. Gluten is present in grains that are traditionally transformed into flour for pasta production, such as wheat, as well as in rye and barley [23]. Hence, alternatives have been developed for these segments of consumers, utilising different flours derived from products that do not contain gluten, such as cowpea, corn, oat, rice, or buckwheat, among others [24,25,26,27,28,29,30]. Buckwheat is an ancient pseudo-cereal with nutritive, functional, and health-promoting properties, which has been gaining relevance and economic importance [31]. It is rich in flavonoids with antioxidant and antidiabetic properties. Furthermore, it has been confirmed as having analgesic, anti-inflammatory, antipyretic, and antiproliferative activities [31,32]. Several studies have successfully incorporated buckwheat into pasta, although this is mostly done in combination with wheat flour [33,34,35,36]. However, there are a few works that specifically used buckwheat flour for the production of gluten-free pasta [29,30].

Although very relevant as alternative flours for gluten-free pasta products, there are multiple technological and sensorial challenges associated with the absence of gluten and the proper development of pasta’s rheological properties, which in turn influence the processing operations of pasta, like moulding, drying, and cooking, as well as organoleptic properties, like texture, colour, or flavour [23,25,37,38].

In the scientific literature there are many studies about development of pasta with improved formulations, including a variety of ingredients like bee pollen [1], chestnut [1], sesame [11], turmeric [39], seaweed [40], millet [41], lentil [41], chickpea [42], broccoli sprouts [43], and mushroom [5], among many others. However, only two works have been found in the literature that use carrot-based ingredients [44,45], but even in these cases, the pasta was formulated using carrot pomace, which is a by-product obtained during carrot juice processing. As such, to the best of our knowledge, there is no work focusing on pasta enrichment that uses carrot as the bioactive ingredient. Hence, the objective of this work was to develop pasta products with and without gluten, using wheat and buckwheat flours, and to incorporate dehydrated carrot in variable proportions. Furthermore, we aimed to conduct a thorough examination of their physical, rheological, chemical, and sensorial characteristics to determine consumer acceptability and nutritional enhancement.

## 2. Materials and Methods

### 2.1. Preparation of the Carrot Powder

Carrots were purchased from a local supermarket, peeled, and cut into slices with a thickness of 3 mm. Then, they were placed in trays with a bottom made of thin metal net for easy circulation of the hot air when in the drying chamber. Convective drying was conducted at a constant temperature of 50 °C for 7 h in a drying chamber, model WTB (Binder GmbH, Tuttlingen, Germany) with an air flow rate of 0.5 m/s. The drying of carrots has been previously studied for convective drying between a range of temperatures from 50 °C to 70 °C, with the drying at 50 °C being faster and more efficient [46]. Additionally, studies recommend using mild temperatures to prevent thermal degradation of carotenoids and other compounds with antioxidant properties, such as phenolic compounds [47,48,49].

**Figure 1:**
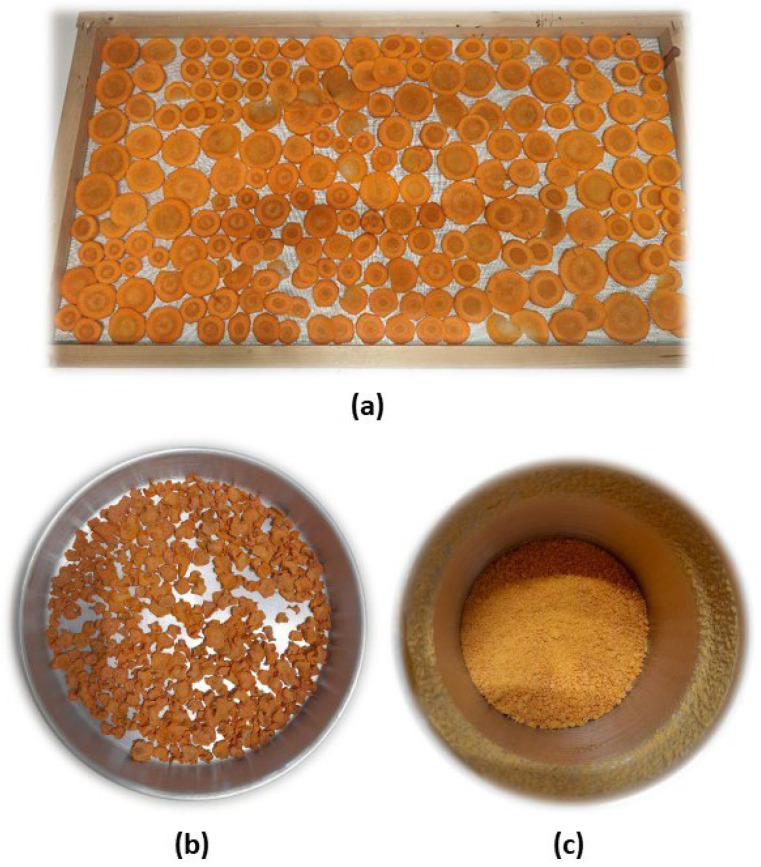
Preparation of the carrot powder: (**a**) before drying, (**b**) after drying, and (**c**) after grinding.

After drying, the carrot slices were ground in a grinder model Q.5321, with a capacity of 60 g and a power of 220 W (from Qilive by Auchhan, Croix, France) (Figure 1).

The final moisture of the dehydrated carrot slices was measured using a Halogen moisture analyser, model HG53 (from Mettler Toledo, Columbus, OH, USA). The operating parameters were 120 °C and evaporation rate = 4 (scale from 1 = very fast to 5 = very slow). The moisture content was analysed in three replicates, and the mean value and standard deviation were then calculated as 5.54 ± 0.16 g/100 g sample (wet basis).

### 2.2. Formulations

Different formulations were prepared, some using wheat flour and others using buckwheat flour, to obtain a gluten-free version of the pasta. For the preparation of the samples, formulations with 0%, 5%, and 10% carrot powder were prepared. This percentage was calculated based on the mass of flour as the reference (0%—100 g of flour + 0 g carrot powder, 5%—95 g of flour + 5 g carrot powder, and 10%—90 g of flour + 10 g carrot powder). We established a limit of 10% because carrot is a rather sweet vegetable and adding a high amount could affect the product, which was not intended to taste sweet. The exact formulations, along with the amounts of each ingredient, are presented in Table 1. Slight variations were due to convenience; for example, one egg was always used, they were supposedly of the same size, but their weights varied. The wheat flour used was type 55 of the brand *Nacional*, the buckwheat flour was of the brand *Cozinha*, the olive oil was extra-virgin of the brand *Oliveira da Serra*, and the eggs were class L (large) of the brand *Matinados*, all of which were on sale at a local supermarket.

**Figure 2:**
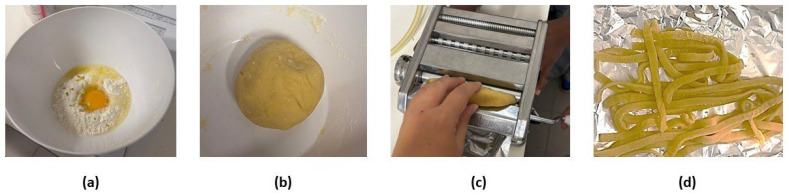
Preparation of the pasta: (**a**) mixing the ingredients, (**b**) dough, (**c**) moulding the sheets, and (**d**) pasta strips.

To prepare the pasta, the ingredients were mixed until a desired consistency was achieved. No water was added to any of the pasta formulations, so that the moisture of the dough resulted only from the added olive oil and egg, which are liquid ingredients. After preparing the dough, it was left to stand for 30 min in the refrigerator before being moulded into pasta strips using a Pasta Moulding Machine PM0500 (LAICA, Barbarano Mossano, Italy). First, the dough was moulded into sheets, and only after were these were cut into strips (Figure 2). In all cases, the samples were analysed in the fresh state and also after dehydration.

### 2.3. Drying of the Pasta

To dry the pasta, the samples were placed in a drying chamber (model FD 115, Binder GmbH, Tuttlingen, Germany) for approximately 4 h at a temperature of 60 °C. Variable temperatures have been reported in the literature for drying pasta from 50 °C [50] to 90 °C [51]. We opted to use the temperature of 60 °C, which was used to dry artisanal pasta in the work of Silva et al. [52], because this temperature corresponds to a compromise between fast drying and a relatively mild temperature that would not degrade the bioactive compounds present, especially phenolic compounds and carotenoids [53,54,55]. The final moisture content was determined using the Halogen moisture analyser (Mettler Toledo, Columbus, OH, USA), with a temperature of 120 °C and a rate of 4. Mean values and standard deviation were calculated from three replicates. Drying loss, also sometimes designated by weight loss, was calculated by the following equation:

(1) $$\mathrm{Drying}\;\mathrm{loss}\;(\%)\;=\;\frac{\mathrm{Weight}\;\mathrm{before}\;\mathrm{drying}-\mathrm{weight}\;\mathrm{after}\;\mathrm{drying}}{\mathrm{Weight}\;\mathrm{before}\;\mathrm{drying}}\times 100\;$$

### 2.4. Cooking of the Pasta

Both versions of the pasta, fresh and dried, were submitted to cooking, and some parameters that characterise the cooking operation were evaluated.

The optimal cooking time was determined by adapting the method described by Chetrariu and Dabija [56]. Twenty grams of pasta were placed in a flask with 200 mL of boiling water, without the addition of salt. At intervals of 30 s, a sample was taken out to evaluate its cooking state by pressing it between two glass plates.

Cooking yield, also referred to as weight increase index, was determined according to the following equation [1,57]:

(2) $$\mathrm{Cooking}\;\mathrm{yield}\;(\%)\;=\;\frac{\mathrm{Weight}\;\mathrm{after}\;\mathrm{cooking}}{\mathrm{Weight}\;\mathrm{before}\;\mathrm{cooking}}\times 100$$

Water absorption was calculated by the following equation [56]:

(3) $$\mathrm{Water}\;\mathrm{absorption}\;(\%)\;=\;\frac{\mathrm{Weight}\;\mathrm{after}\;\mathrm{cooking}-\mathrm{Weight}\;\mathrm{before}\;\mathrm{cooking}}{\mathrm{Weight}\;\mathrm{before}\;\mathrm{cooking}}\times 100$$

Cooking loss was determined according to established methodologies [56,58], based on the weight of the residue left after cooking with the evaporation of all the water, thus accounting for the mass of pasta that was lost into the boiling water during the cooking procedure. For this, 20 g of fresh pasta was cooked in 200 mL of boiling water until the optimal cooking time was reached. The cooking water was then placed into a weighted flask and left in an oven set at 110 °C to dry until complete evaporation. The flask was then weighed, and the difference was calculated corresponding to the mass of residue. Cooking loss is expressed as a percentage of the initial mass of pasta according to the following equation [59]:

(4) $$\mathrm{Cooking}\;\mathrm{loss}\;(\%)\;=\;\frac{\mathrm{Weight}\;\mathrm{of}\;\mathrm{cooking}\;\mathrm{residue}}{\mathrm{Weight}\;\mathrm{of}\;\mathrm{uncooked}\;\mathrm{pasta}}\times 100$$

### 2.5. Hydration Test

A hydration test was performed on all dried samples, following an adaptation of the procedure described by Schettino et al. [60] and by Chetrariu and Dabija [56]. For this, 1 g of each pasta sample was placed into separate beakers filled with 20 mL of tap water. The beakers were left in a thermostatic chamber set at 25 °C for incubation. Samples were removed after 10, 20, 30, 60, 90, 120, and 150 min, drained for 1 min, and then carefully wrapped with tissue paper to remove just the superficial water before weighing. The hydration over time was calculated according to the following equation, for all times, i.e., from t = 0 until t = 150 min [56,60]:

(5) $$\mathrm{Hydration}\;(\mathrm{at}\;\mathrm{time}\;\mathrm{ti})\;(\%)\;=\;\frac{\mathrm{Weight}\;\mathrm{of}\;\mathrm{hydrated}\;\mathrm{sample}\;\mathrm{at}\;\mathrm{ti}}{\mathrm{Weight}\;\mathrm{of}\;\mathrm{the}\;\mathrm{dry}\;\mathrm{sample}}\times 100$$

### 2.6. Biometry

The dimensions of the pasta strips were measured with a digital calliper rule with an accuracy of ±0.02 mm and a 5-digit LCD display and measurement length of 150 mm (KNUTH Machine Tools, Wasbek, Germany). Measurements were taken for six different strips of pasta from all samples at various stages (fresh, fresh-cooked, dried, and dried-cooked). Measurements were recorded for width and thickness in each analysed sample.

### 2.7. Colour

The colour of all samples, fresh, dried, and cooked, was determined using a Colourimeter Chroma Meter—CR-400 (Konica Minolta, Tokyo, Japan), which measures the Cartesian coordinates L*, a*, and b*, respectively, for lightness and opposed colour coordinates. Lightness, L*, varies from 0 (black) to 100 (white) and describes all tones of grey. Coordinate a* varies between −60 and +60, corresponding to green when assuming negative values and red when assuming positive values. Coordinate b* also varies between −60 and +60, corresponding to green when negative and yellow when positive. Colour measurements were performed six times for each pasta sample. From the Cartesian coordinates L*, a*, and b*, the total colour difference (TCD) was calculated, with the control sample as reference, using the following equation [1,56]:

(6) $$\mathrm{TCD}\;=\;\sqrt{{\left(L*-L_{0}*\right)}^{2}+{\left(a*-a_{0}*\right)}^{2}+{\left(b*-b_{0}*\right)}^{2}}$$

The control sample considered was pasta made with wheat flour and no added carrot powder (WG0), at the different stages examined (fresh, dried, and cooked). Khan et al. [61] present the limits for interpretation of the TCD as follows:

- 0.0–0.5: trace difference;
- 0.5–1.5: slightly noticeable, but detectable with the human eye;
- 1.5–3.0: noticeable difference, but detectable by a trained observer;
- 3.0–6.0: appreciable, detectable by regular people;
- 6.0–12.0: large difference, but within the same colour category;
- >12.0: extreme, i.e., a difference that corresponds to another colour category.

### 2.8. Texture

**Figure 3:**
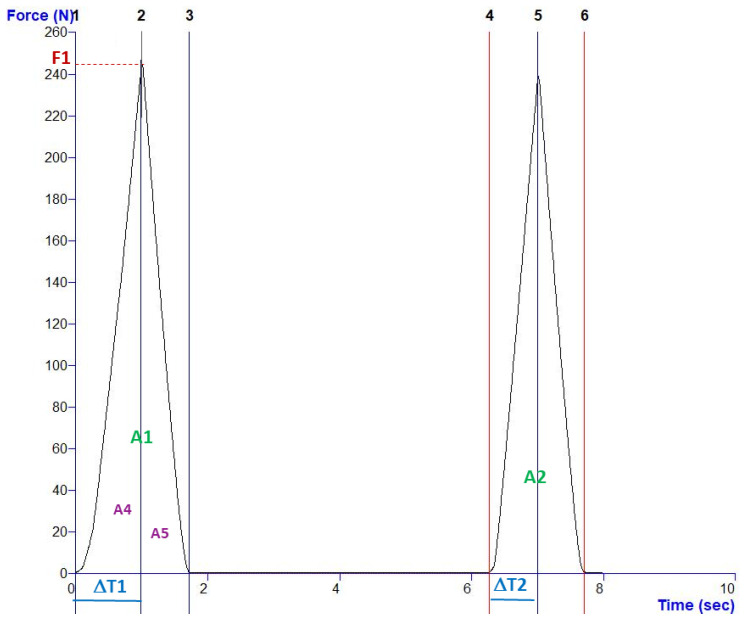
Example of a curve obtained for the determination of the textural parameters.

Texture profile analysis (TPA) was performed on six strips of pasta for each sample at various stages (fresh, fresh-cooked, dried, and dried-cooked). A texturometer, model TA.XT.Plus (Stable Micro Systems: Godalming, Surrey, UK), was used, equipped with a 50 kg load cell. A cylindrical probe (P/75) was used to perform two consecutive compressions, separated by an interval of 5 s. The test parameters were as follows: the pre-test, test, and post-test speeds were all set to 1.0 mm/s, the compression distance was 1 mm, and a trigger force of 0.5 N was applied. The analyses produced curves similar to that in Figure 3, which corresponds to one TPA obtained for sample WG5-Dried (pasta with gluten and 5% carrot powder after drying), is an example to explain how the textural parameters were calculated, according to the following equations [62,63]:

Hardness (N) = F1(7)

(8) $$\mathrm{Resilience}\;(\%)=\frac{A5}{A4}\times 100$$

(9) $$\mathrm{Cohesiveness}\;(\%)=\frac{A2}{A1}\times 100$$

(10) $$\mathrm{Springiness}\;(\%)=\frac{∆T2}{∆T1}\times 100$$

(11) $$\mathrm{Chewiness}\;(N)=F1\times \frac{∆T2}{∆T1}\times \frac{A2}{A1}$$

### 2.9. Pasting Properties

The pasting properties of the pasta samples were assessed using a Rapid Visco Analyser—RVA 4500 (Perten, Stockholm, Sweden). The RVA is a viscometer that heats and cools the sample according to an established program, measuring the viscosity of the sample under stirring over a specified period of time [64]. This methodology enables continuous evaluation of the sample’s viscosity under variable temperature conditions, registering the effect of temperature on viscosity in the form of a continuous curve. The RVA measurements for the dried uncooked pasta samples were adapted from Güller et al. [58] and Gull et al. [65]. For the analyses, 3.5 g of previously ground pasta was added to 25 mL of distilled water, and the tests were run according to the programs presented in Table 2. Since the nature of the starches differs depending on the presence or absence of gluten, adjustments were made to the temperature profile of both tests, specifically with a lower starting temperature for the gluten-free pasta [66].

**Figure 4:**
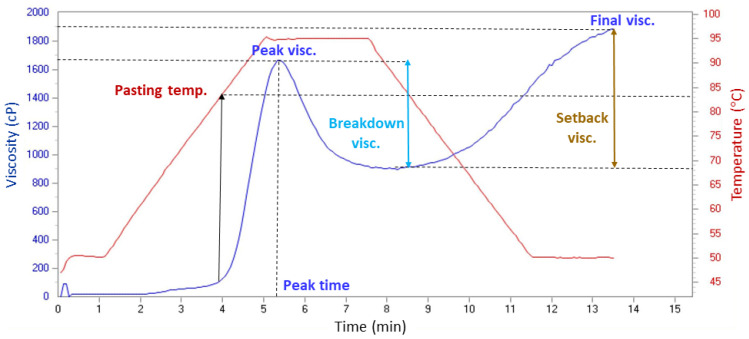
Example of a curve obtained for the determination of the pasting properties.

The rheological parameters measured during the test were as follows: pasting temperature, peak viscosity, peak time, breakdown viscosity, final viscosity, and setback viscosity (as shown in Figure 4). The RVA measurements were performed in triplicate.

### 2.10. Chemical Analyses

The dried, cooked pasta samples were triturated in a grinder, model Q.5321 (Qilive by Auchhan, Croix, France), and analysed for composition by NIR (near-infrared) Spectroscopy [34] using a spectrometer, model NIRMaster (BUCHI, Barcelona, Spain). The parameters analysed for centesimal composition were moisture, fat, protein, and ash, with the total carbohydrates calculated by difference, subtracting the sum of moisture, protein, fat, and ash from 100 [65]. Additionally, the NIR Spectroscopy also determined the presence of starch, lysine, cystine, methionine, and phosphorus. All measurements were made in three replicates.

For the determination of carotenoids, extracts were produced as follows: 5 g of each dried sample was mixed with 50 mL of an acetone–hexane solution (4:6) using a magnetic stirrer for 10 min. These extracts were then filtered and separated into two parts. One was used to determine lycopene and beta-carotene, and the other was incubated for 5 min at 37 °C for the determination of total carotenoids. Absorbance was measured in a spectrophotometer, model E-1000UV (Peak Instruments, Shanghai, China). For total carotenoids, absorbances were read at 435, 505, and 663 nm, while for lycopene and beta-carotene, absorbances were read at 453, 505, 645, and 663 nm [67,68]. Calculations followed the formulas below:

Total carotenoids = 0.2165 × A_663_ − 0.304 × A_505_ + 0.452 × A_453_ (12)

Lycopene = −0.0458 × A_663_ + 0.204 × A_654_ + 0.372 × A_505_ − 0.0806 × A_453_ (13)

Beta-carotene = 0.216 × A_663_ − 1.22 × A_654_ − 0.304 × A_505_ + 0.452 × A_453_ (14)

All measurements were made in triplicate and expressed in mg/g but were later converted to mg/100 g dry weight.

Total phenolic compounds were determined after extraction with a mixture of ethanol and water. For extraction, an adaptation of the method described by Schettino et al. [60] was used. To a mass of 5 g from each sample, 25 mL of ethanol and 25 mL of water were added, and the mixture was left for 1 h at 28 °C with magnetic stirring at 150 rpm. After that, the samples were centrifuged at 3000 rpm for 13 min in a centrifuge, model MPW-260R (MPW Med. Instruments, Warsaw, Poland), and the supernatant was separated for spectrophotometric analysis.

To analyse the phenolic compounds content, the Folin–Ciocalteau method was used [57,65]. The extracts obtained were diluted for analysis (1:4, extract–solvent mixture of ethanol and water, 50%). To a tube were added 125 µL of the diluted extracts, 750 µL of distilled water, and 125 µL of Folin reagent, and it was left to stand for 6 min. Then, 2000 µL of sodium carbonate solution (5%) was added and left in the dark at room temperature for 60 min. Absorbance was read at 670 nm in a Spectrophotometer, model E-1000UV (Peak Instruments, Shanghai, China). Measurements were made in triplicate and were expressed in Gallic Acid Equivalents (GAE).

### 2.11. Sensory Analysis

The assessment of the sensory characteristics was made based on the procedures described by El-Sohaimy et al. [63] and Torres et al. [69]. The sensory analysis took place in the Laboratory of the Food Industry Department at the Agrarian School of Viseu, Portugal. A panel consisting of 48 members (35 women and 13 men, with ages ranging from 18 to 57 years) evaluated the six samples of pasta after the drying and cooking operations had been carried out. Cooking of the pasta samples was performed at optimal cooking time, without the addition of salt. All samples (three with gluten—made with wheat flour, and three gluten-free samples—made with buckwheat flour), with variable percentages of carrot powder (between 0 and 10%), were presented to the panellists with a code that made it impossible to reveal the pasta characteristics or formulations. The panellists were instructed to taste the samples and rinse the mouth in between each sample for optimal assessment. They were asked to rate the samples according to their acceptance relative to the following sensorial attributes: visual aspect, mouthfeel, taste, aroma, colour, and texture. The measuring scale was a 9-point hedonic scale varying from 1 = extremely unacceptable to 9 = extremely acceptable.

### 2.12. Data Treatment and Statistical Analysis

To evaluate whether the results obtained in terms of mean value were statistically different among the pasta samples, statistical analysis was performed on the data obtained for the pasta properties, including chemical analyses, colour, texture, and sensory analysis.

A post hoc Tukey HSD (honestly significant difference) test was used, coupled to an analysis of variance (ANOVA) to compare the mean values between the six types of pasta. Tukey’s test is a statistical method used to identify differences among groups of data, which is carried out in conjunction with the ANOVA. This allows for the identification of which samples differ from one another by locating where the difference between two mean values is higher than the expected standard error [1].

All statistical tests were made using the software SPSS version 29 (IBM, Inc., Armonk, NY, USA), considering a level of significance of 5% (*p* < 0.05). For drawing graphs, we used Excel from Office 2016 (Microsoft, Redmond, WA, USA).

## 3. Results and Discussion

### 3.1. Preparation, Drying, and Hydration Characteristics of the Pasta Samples

The preparation of the different versions of pasta, with and without gluten, and with variable percentages of carrot powder, followed the formulations indicated in Table 1, previously presented in the Materials and Methods Section. The upper limit established for the proportion of carrot powder was 10% because carrot is a rather sweet vegetable, which is beneficial for some food applications, such as vegetable drinks or even to mix with fruit or dairy drinks [70,71,72], but not so much for others, where they are not intended to taste sweet. Hence, it was not aimed to produce a pasta that would have a sweet flavour, and the addition of a high amount of carrot powder could possibly affect the product’s organoleptic characteristics in a negative way.

It is important to note that during handling, it was observed that formulations made with wheat flour were easier to work with, more consistent, and more elastic when compared with samples containing buckwheat flour, allowing for the production of gluten-free versions of the pastas. This resulted in a greater difficulty in handling the gluten-free pastas, not only when moulding the strips, but also when cooking the pasta. This is expected since the dough-forming properties of wheat flour are significantly enhanced compared to buckwheat, making it suitable for the production of bakery products. A study by Coţovanu et al. [73] evaluated the effect of partial replacement of up to 20% of wheat flour by buckwheat flour for the production of bread and observed a noteworthy difference during bread-making operations as well as on the final products. Increasing the percentage of buckwheat flour induced significant changes in dough development time, the rate of protein weakening, alveograph ratio (the balance between dough tenacity and extensibility), rheofermentation properties, gel starch stability, maximum creep-recovery compliance, and bread firmness [73]. Nevertheless, buckwheat is considered a valuable ingredient for obtaining bakery products with high nutritional value [74,75] and is especially suitable for diets free of gluten, such as those for individuals with celiac disease or gluten intolerance. People with celiac disease can use buckwheat as a substitute for wheat-based cereals, although buckwheat is also suitable for other dietary needs due to richness in various nutrients [76,77].

Although it is possible to consume fresh pasta in some restaurants or prepare it at home, the majority of pasta is more commonly consumed after drying, due to the many advantages it offers. Dried pasta is easier to transport due to its lighter weight, high shelf life, and because it does not require refrigeration. Table 3 presents the drying parameters for the six formulations tested.

The results in Table 3 showed that drying loss was smaller for the pasta samples made with wheat flour (versions with gluten) than for the samples made with buckwheat (gluten-free versions). The values of drying loss obtained for the six pasta samples were between 20 and 30%, which were lower than those observed by Brochard et al. [1] for pastas incorporating bee pollen and chestnut flour (range between 57% and 67%). It is important to note that in the present work, the drying operation was carried out at 60 °C for a period of 4 h, while in the work by Brochard et al. [1], the drying of the pasta samples was carried out at 40 °C for a more extended period of 12 h. The higher temperature enabled a faster and more efficient extraction of moisture from the samples, achieving a very low moisture content, as indicated by the final values, below 1% for the gluten versions and below 1.5% for the gluten-free versions (Table 3). For the dried pasta to be safely stored, its moisture content must be below 12% [56,78]; hence, the produced samples contain a moisture content that is suitable for storage.

**Figure 5:**
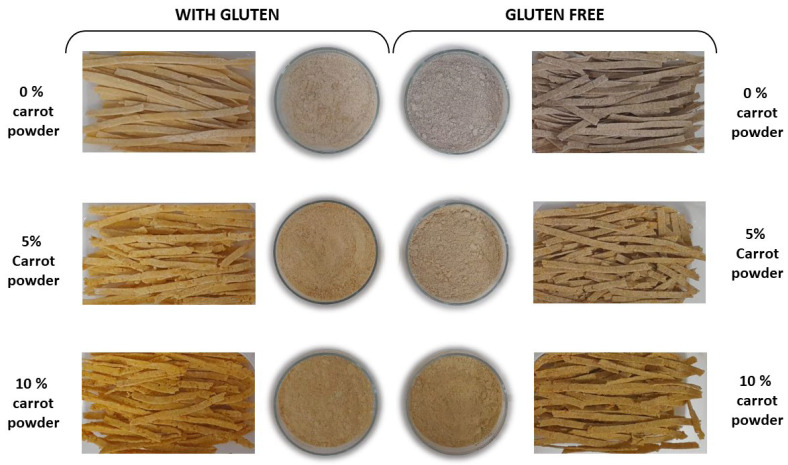
Dried pasta samples.

Figure 5 presents the six samples of pasta after drying, highlighting the changes according to the type of flour used and the percentage of carrot powder used, particularly in terms of colour, which is also observable when the dried pasta is ground.

**Figure 6:**
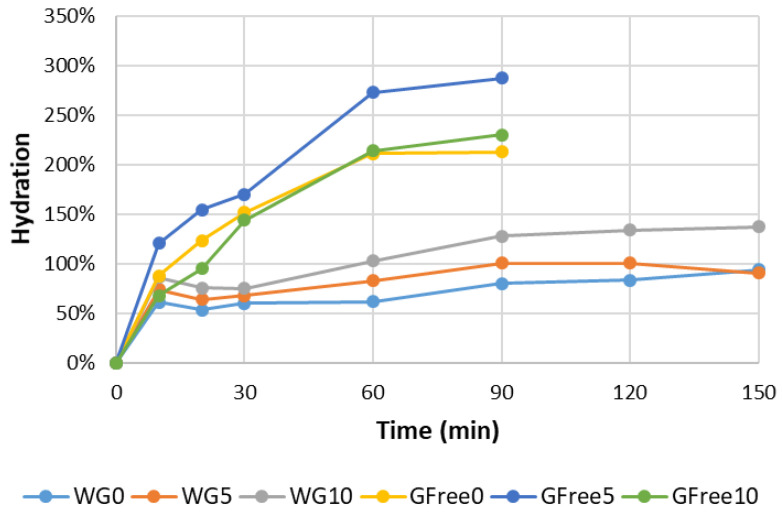
Hydration of the dried pasta samples.

Dried pasta is consumed after rehydration, which typically occurs during the cooking process. However, the hydration test is also important to study how the dried samples behave in the presence of moisture. In fact, all dried foods tend to be hygroscopic, absorbing moisture due to their low water content. Hence, it is essential to investigate the processes occurring during the rehydration of dried pasta, which involve complex mass transport phenomena governed by various migration mechanisms of water into the pores [63]. Figure 6 presents the rehydration profiles of the six pasta samples under study, as obtained by the rehydration test. The data stops at 90 min for the gluten-free versions because, at this stage, these samples were no longer viable, i.e., they had disintegrated in the water. Conversely, the gluten versions maintained their integrity in the water until the end of the test (2 h and 30 min). The results further showed that the water uptake during the hydration test was significantly higher for the gluten-free samples (between 200% and 300%) than for the gluten-containing versions (below 150%). In the work by Schettino et al. [60], the hydration of pasta samples was lower, between 60% and 100%, for pastas made with wheat flour and incorporating brewer’s spent grain, in a hydration test conducted under similar conditions (ratio sample/water and temperature). The type of proteins and the presence/absence of gluten influence the hydration characteristics of pasta [56].

### 3.2. Cooking Properties of the Dried Pasta

The results in Table 4 indicate that the optimal cooking time was shorter for the gluten-free pastas (containing buckwheat) compared to the wheat-based pasta samples. Additionally, the dried pastas required less time to reach the optimal cooking point compared to their fresh counterparts. The cooking time of the pasta samples analysed in this study varied in the range of 6 m 30 s–10 m 0 s, which is similar to the optimal cooking times obtained by Gull et al. [65] for durum semolina pasta (7 min) and millet–pomace-based pasta (6 min). While Torres et al. [69] found higher cooking times for semolina pastas supplemented with lupine flours (varying between 8 and 15 min.), El-Sohaimy et al. [63] found lower values of optimal cooking time for pastas fortified with chickpea (varying from 5 min. to 6.5 min.). These results indicate that the optimal cooking time is highly influenced by the formulation, specifically, the ingredients used in its production. According to El-Sohaimy et al. [63], the optimal cooking time varies inversely with the protein content. A decrease in cooking time can be supported by an increase in the rate of water migration into the pasta core. This can be attributed to the lack of continuity in the protein–starch network, which allows water to diffuse more easily through the pasta matrix. As such, the time necessary for the water to reach the centre of the pasta during the cooking operation is lower. The augmented water penetration is attributed to the physical disruption of the gluten matrix, which provides a path for water absorption into the sample [79,80].

The cooking yield (Table 4), reflecting the weight gain during the cooking operation, was considerably higher for the dried samples. One reason that could account for this difference is the significantly lower moisture content at the beginning of the cooking operation for the dried pasta samples, resulting in a higher concentration gradient and a faster water diffusion rate. On the other hand, according to Brochard et al. [1], the differences in cooking yield between fresh and dried pasta can be attributed to changes occurring during the drying operation in the ingredient’s components, namely protein and protein–starch interactions. The cooking yield varied in the ranges of 171–197% and 228–289%, respectively, for the fresh and dried pastas. These ranges are very similar to those obtained by Brochard et al. [1] for pasta samples containing bee pollen and chestnut flour, which were 179–123% and 201–278% for fresh and dried pastas, respectively. It was further observed that there was a trend towards higher cooking yields in the buckwheat pasta samples compared to the wheat pasta samples. Finally, the trends observed for cooking yield and water absorption are very similar, as both relate to water uptake during the cooking operation. Changes detected between samples containing buckwheat and wheat flours were expected since the gain in weight observed for the cooked pasta samples relates to the water absorption resulting from a complex molecular modification of starch and proteins, mainly in hydration, and the composition of the pastas regarding the starch and proteins is different for buckwheat and wheat flours [59].

Cooking loss varied between 2.42% and 5.08% for the fresh pasta samples and was slightly higher, between 3.53% and 6.31%, for the dried samples (Table 4). These values were lower compared to those reported by Gull et al. [65] for durum semolina and millet–pomace-based pastas, 7.66% and 6.10%, respectively. A slight cooking loss indicates a well-developed protein network, which is one of the most important characteristics that determine consumer acceptance [56,60,81]. Differences have been reported in the cooking loss of wheat-based and gluten-free pastas due to variations in the protein–starch networks [65,82,83]. A higher protein content has been associated with a negative effect on gluten development, making the structure more fragile and thus facilitating the release of solids, which increases cooking loss [63,84]. Torres et al. [69] observed that increasing the percentage of lupine flour in semolina-based pasta formulations led to an increase in cooking loss.

### 3.3. Dimensions of the Pasta Samples

The biometric characteristics of the pasta samples were measured at different stages (fresh, fresh-cooked, dried, and dried-cooked), for all six types of pasta, with the results presented in Table 5. The results show that cooking increases both dimensions (width and thickness) for both types of pasta (fresh and dried), due to the water uptake that occurs during the cooking process. The observed differences were statistically significant, as can be seen in Table 5.

### 3.4. Colour Properties of the Pasta Samples

Colour is an extremely important factor determining the visual quality, consumer acceptance, and market value of food products [65]. Table 6 presents the colour characteristics of the six pasta formulations (with and without gluten and with varying percentages of carrot powder) at different stages (fresh, fresh-cooked, dried, and dried-cooked). The results indicate a trend for higher lightness (L*) in the wheat-based pastas at all stages, compared to the buckwheat-based pastas, which tend to be darker, with significant differences as indicated by the statistical analysis results. Additionally, it was observed that increasing the percentage of carrot powder led to a statistically significant increase in the colour coordinate a* (redness, with positive values), except for the dried versions of the gluten-free samples. Furthermore, the b* coordinate showed significantly higher values for the wheat-based pastas as compared to the buckwheat pastas, which presented a less yellow colouration. Kumar et al. [78] reported that typical wheat pasta tends to possess high lightness and low redness, which corresponds to sample WG0 in this study.

Regarding the values of total colour difference (Table 6), they corresponded in most cases to extreme differences (TCD > 12), i.e., they correspond to different colour categories, as described by Khan et al. [61]. Only the WG5 fresh and GFree0 dried samples have values of TCDs lower than 12, and even in this case, they corresponded to large differences, although they are within the same colour category [61].

Regarding the variations in colour that occur during the different stages of pasta production, it was observed that the cooking operation tends to decrease the lightness, resulting in darker samples (both for fresh and dried pasta), possibly due to the effect of temperature. These differences were statistically significant. Gull et al. [65] observed a contradictory trend, i.e., the cooking operation leading to an increase in L*, possibly due to colour loss during the cooking process. Cooking also led to a reduction in redness (lower a*), likely due to the thermal degradation of carotenoids and consequent transfer of some components into the cooking water. Due to the highly unsaturated structures of carotenoids, they are susceptible to oxidation, isomerisation, and degradation, which can be caused by exposure to heat, light, and oxygen, among other factors [85]. Similar results for loss of carotenoids in cooking were obtained by Gull et al. [65] for pasta samples containing carrot pomace. According to the same authors [65], the colour changes observed can be attributed to the swelling and transformation of pigments occurring during the cooking process.

**Figure 7:**
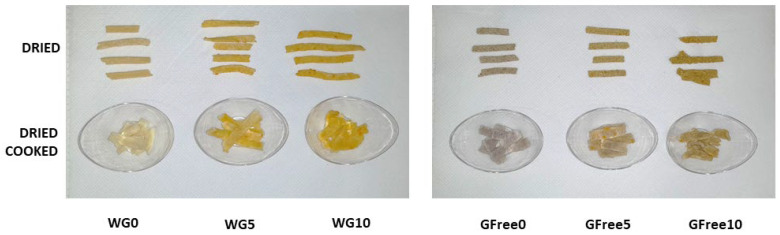
Dried pasta samples, before and after cooking.

For commercialisation purposes and the general consumer, the dried pasta is the most important, as the dried form constitutes the best way to safely transport and store the pasta from industrial plants to the final consumer, who will then cook it for consumption. For this reason, the dried and dried-cooked pasta samples are of particular importance [56], and are shown in Figure 7, corresponding to the six different formulations produced in the present work. The addition of increasing percentages of carrot powder resulted in more pronounced orange colouration, particularly in the case of the wheat-based pastas. Additionally, it can be seen that the trends are similar regardless of whether they are for the dried or dried-cooked pasta samples. It is visible that the buckwheat-based pasta exhibits a high differentiation in relation to the wheat-based pasta without the addition of carrot powder, indicating that the nature of the sample is responsible for a high colour difference. In fact, the colour of pasta is highly variable, depending on the ingredients used, as well as technological processing [57]. In general, brightness and yellowness are considered important colour characteristics for both consumers and manufacturers, as a bright yellow colour is often associated with high-quality pasta. However, enrichment of pasta with different ingredients frequently results in colour changes [57]. Still, the consumer is often attracted to enriched products due to their improved nutritional profile, even though they may have different colours. For individuals who cannot consume wheat-containing pastas, such as those with celiac disease or gluten intolerance, it is essential to seek alternatives made with gluten-free ingredients, like buckwheat, which is used in the present work. The colour of buckwheat is very different from that of wheat flour [86].

### 3.5. Pasting and Textural Properties of the Pasta Samples

During pasting, a complete disruption of the starch granules occurs at a temperature higher than that of gelatinisation. Hence, pasting causes the development of a viscous gel, with variable viscosity according to rheological principles [64]. Table 7 presents the pasting properties of the six formulations of dried uncooked pasta. It is well established that the viscosity of starch undergoes modifications during heat processing, such as those occurring during the RVA test. This is due to the swelling of starch granules, the loss of crystallinity in amylopectin and the leaching of the amylose into the water [64,66]. The pasting temperature corresponds to the value when the viscosity of the starch begins to increase [64] (Figure 4). The results showed that the pasting temperature was higher for the buckwheat-based pastas than for the wheat-based pastas and increased with the increasing percentage of incorporated carrot powder, although at a more pronounced rate for the samples containing gluten.

Peak time, which corresponds to the moment when the peak viscosity is achieved, showed different trends according to the type of pasta, increasing for the wheat-based pastas with an increased percentage of carrot powder, but decreasing as the percentage of carrot powder increased for the gluten-free samples.

The peak viscosity, which represents the shear resistance of particles [65], increased with increasing percentage of carrot powder in the samples containing wheat, and was higher for the buckwheat-based pastas as compared to the wheat-based ones. The peak viscosity reflects the capacity of the starch granules to swell spontaneously before they breakdown, i.e., before their physical integrity is compromised [65]. The peak viscosity is directly proportional to the starch gelatinisation intensity [66], and therefore, different flours with different starch compositions have different behaviours. The swelling at the beginning of the gelatinisation process, which occurs at lower temperatures, may be higher in the presence of carrot powder, resulting in a greater increase in viscosity [66]. It has also been reported that the presence of hydrocolloids in the formulations, which bind water and increase volume, allows increasing viscosity with a small degree of swelling of the starch granules [66]. On the other hand, starch damage can result in a lower swelling capacity and hydration of granules, thus leading to a reduced pasting viscosity [58]. Also, the protein content of the different pasta samples can be inversely related to peak viscosity [65].

The breakdown viscosity, corresponding to the difference between the peak and trough (Figure 4), is linked to the stability of hot starch pastes [65]. This showed a trend of increasing with the increase in the carrot powder ratio for the wheat-based samples, while decreasing for the buckwheat-based samples. The addition of carrot powder to the wheat-based pasta affected stability, as indicated by the higher breakdown values.

The final viscosity corresponds to the value at the end of the test [65] (this time being variable according to the program defined to wheat-based or buckwheat-based pastas, respectively, 13 m 30 s and 11 m 30 s) (Table 2, in the Materials and Methods Section). The results showed that consistently higher values were obtained for the samples containing 5% carrot powder, for both types of pasta (wheat-based and buckwheat-based). It was also observed that higher values of final viscosity were found for the gluten-free pastas than for the pastas containing gluten.

Lastly, setback viscosity, which represents the difference between the final and through values [65], was once more higher for the buckwheat-based pastas than for the wheat-based ones. Regarding the variation according to the percentage of incorporated carrot powder, it showed a similar trend to the final viscosity, i.e., being higher for the samples containing 5% carrot. Setback viscosity is a measure of the retrogradation and reordering of starch molecules. Hence, lower values of setback correspond to lower rates of starch retrogradation and syneresis, indicating an improved quality of the pasta [65]. The results showed that, for the study samples, the higher the percentage of added carrot powder, the better the quality of the pasta.

According to Güler et al. [58], although a small part of the starch can be damaged during the extrusion phase when moulding the pasta strips, in reality, the greatest change occurs during the drying operation, when the amylolytic enzymes present can act on the starch that is mechanically damaged.

Table 8 presents the results obtained for the textural parameters measured in all pasta samples and at various stages of processing. The obtained results showed that hardness increased with the increased percentage of carrot powder in both variations (with gluten and gluten-free pastas) for the fresh versions of the pasta (fresh and fresh-cooked samples). These results are contrary to those observed by El-Sohaimy et al. [63], who reported a decrease in hardness with an increased percentage of chickpea in wheat-based fresh-cooked pastas. Hardness, also sometimes designated as firmness, corresponds to the force necessary to penetrate the pasta with the teeth and represents the resistance presented by the sample to the first bite, which also associated with the ‘al-dente’ mouthfeel [56]. The gluten-free samples generally showed slightly higher hardness than those with gluten, with significant differences indicated by the statistical analysis results, although there were some exceptions where the opposite was observed. However, according to Kumar et al. [78], the gluten content improves both the firmness and stickiness of wheat-based pastas. On the other hand, the increase in the percentage of added carrot powder resulted in a decrease in hardness for the dried samples and an increase for the fresh samples. A similar increase in hardness for pasta containing added barley was reported by Schettino et al. [60]. The cooked samples showed lower hardness compared to their fresh counterparts, with this effect being more pronounced for the dried-cooked samples in relation to the dried ones. During cooking, the development of the pasta firmness is influenced by the hydration of the starch granules [78]. The interaction of egg lipids with amylose, during pasta cooking, limits its solubility in water [1].

Resilience showed an increasing trend with the addition of carrot powder, which was lower for the gluten-free pasta samples. While drying increased resilience, cooking the pasta increased resilience for the fresh samples but decreased it for the dried samples, with significant differences. The addition of carrot powder slightly increased the cohesiveness of all pasta samples. According to El-Sohaimy et al. [63], fortification of pasta can affect cohesiveness, thereby increasing its value compared to control samples, i.e., those with no added carrot powder. However, other authors did not detect changes in cohesiveness resulting from pasta fortification [60]. When comparing the gluten-free samples with those containing gluten, it was observed that the gluten-free samples were less cohesive. This confirmed what had previously been observed during the moulding of the pasta strips, during the rehydration test, and during the cooking operation.

Textural parameters, such as hardness or cohesiveness, are dependent on the structure of the pasta and relate to the pasting properties. While faster heating causes faster swelling of the granules, during the cooling stage, retrogradation occurs, thus altering the structure. As such, modifications occur in the crystalline structures of starches [64]. Since amorphous and crystalline phases have different hardnesses, it is expected that samples with different pasting behaviour will show a corresponding variability in the textural properties.

Springiness (also known as elasticity), unlike cohesiveness, significantly decreased with the increase in the percentage of added carrot powder, with this trend remaining constant regardless of the pasta state (fresh, dried, or cooked). Comparing the gluten-free samples, they exhibit lower elasticity with significant differences from the gluten versions, irrespective of whether they contain carrot powder or not. These results indicate that the presence of gluten in the pasta results in a matrix with greater elasticity as a consequence of the starch structure. Although observed these differences, some authors did not report changes in springiness according to variable percentages of fortification ingredients [56,60].

Chewiness was significantly higher for the dried samples, which was much influenced by the high values of hardness. This was expected, as the drying operation induces a high degree of water removal, thereby making the structure much harder and more difficult to chew. In relation to the addition of carrot powder, it increased the chewiness of the samples containing gluten, but the same trend was not observed for the gluten-free samples. In fact, the wheat-based pasta microstructure is homogenous, entrapping the proteins in the protein–starch matrix. As such, wheat commonly possesses a compact and thick structure [78].

### 3.6. Chemical Properties of the Pasta Samples

Chemical properties of the dried-cooked pasta samples determined by FT-NIR Spectroscopy are shown in Table 9, and significant differences were registered between the samples for all the chemical properties evaluated. These samples correspond to the pasta as it is consumed, i.e., after the drying and cooking operations are complete, making it suitable for ingestion as a meal element. Although the moisture content is expressed on a wet basis, all other components were converted to a dry basis for easier comparison. The moisture content of the samples ranged from 58.55 ± 2.40% to 67.90 ± 1.05%, and it is directly linked with the cooking properties of the pastas, as previously discussed.

Regarding the fat content, it varied from 11.11 ± 0.12% db to 13.30 ± 0.07% db, with sample GFree10 presenting the highest value. These values are much higher than those reported by Torres et al. [69] (0.1–0.2 g/100 gdb), by Schettino et al. [60] (2.2–3.5 g/100 gdb), by Gull et al. [65] (1.8–6.6 g/100 db), and by Chetrariu and Dabija et al. [56] (6.0–7.2 g/100 gdb), for various pasta samples. The pastas developed in the present work were formulated with egg and olive oil, which adds some fat. Specifically, olive oil is considered a highly healthy source of fat, containing high levels of unsaturated (mono and polyunsaturated) fatty acids and polyphenols, thus possessing high nutritional quality and resistance against oxidation [87]. Furthermore, olive oil is reported to have multiple benefits for human health, including improved cardiovascular health and the prevention of cancer and diabetes [88,89,90].

In what concerns the protein content of the six pasta formulations, the values ranged from 14.99 ± 0.18% db to 18.38 ± 0.21% db, with the richest sample being GFree10. These values are higher than those reported by Schettino et al. [60] (14.1–15.2 g/100 gdb), by Torres et al. [69] (13.5–15.5 g/100 gdb), by Gull et al. [65] (11.2–12.7 g/100 db), and by Chetrariu and Dabija et al. [56] (5.9–9.0% db), for variable pasta samples. The protein content was higher for the samples containing buckwheat than for those containing wheat. Although the proximate composition of buckwheat grain does not differ significantly from that of cereals like wheat, buckwheat protein contains low amounts of alcohol-soluble gluten proteins, thus being a convenient alternative source for cereal-based foods for celiac disease patients [91,92].

Ash content varied in the range 1.41 ± 0.01–2.45 ± 0.11% db, with the highest value for sample GFree10. These values, although lower than those found by Chetrariu and Dabija et al. [56] (3.0–3.3% db), are higher than most values of ash reported in the literature for different pastas by Gull et al. [65] (0.4–0.9 g/100 db), Torres et al. [69] (0.6–1.1 g/100 gdb), or Schettino et al. [60] (1.0–1.6 g/100 gdb).

Carbohydrates were the major component in the pasta samples, varying from 65.87 ± 0.28% db to 72.21 ± 0.13% db, with the lowest value for sample GFree10. Our values are within the same range as those reported by Schettino et al. [60] (65.7–79.4 g/100 g db), although other authors reported higher carbohydrate contents in pasta samples, such asGull et al. [65] (77.9–82.6 g/100 g db) and Chetrariu and Dabija et al. [56] (93.7–97.6% db).

Starch was found to range from 55.70 ± 0.62% db to 64.08 ± 0.24% db, being lower than the contents reported by Torres et al. [69] (71.1–74.3 g/100 g db). It was further observed that a decrease in the starch content occurred as the percentage of carrot powder increased, due to a reduction in the wheat and buckwheat flour. Finally, it was observed that the starch content was higher in the wheat-based pastas as compared with the buckwheat counterparts.

The contents in lysine varied from 1.30 ± 0.06 to 1.60 ± 0.02% db, in cysteine varied from 0.45 ± 0.01 to 0.65 ± 0.01% db, and in methionine varied from 0.23 ± 0.00 to 0.27 ± 0.00% db. The values reported by Schettino et al. [60] were expressed as relative percentages and varied in the ranges of 21.4–28.6%, 55.7–73.7%, and 48.0–51.5%, respectively, for lysine, cysteine, and methionine in pasta samples containing brewer’s spent grain. These results report higher relative fractions of cysteine and methionine compared to lysine, which is an opposing trend to what was observed in our pasta samples, where lysine was the most abundant amino acid present.

The amounts of lysine, cysteine, and methionine, although low (<2% db, <1% db, and <0.5% db, respectively), are relevant for numerous functions in the human body. It is observed that the values of the amino acids tested were higher in the samples containing buckwheat than in the samples formulated with wheat flour. In fact, buckwheat is an ancient pseudo-cereal rich in protein and contains high amounts of essential amino acids (such as lysine and methionine) that are not present in some other cereal crops [93]. Lysine is essential for protein synthesis, tissue repair, enhancing muscle and bone strength, producing collagen, and alleviating anxiety and stress. It is also helpful in preventing herpes flares and regulating blood pressure [94,95,96,97,98]. Methionine also plays a crucial role in protein synthesis and antioxidant production, being essential for metabolism, DNA activity, and the production of neurotransmitters and hormones. Additionally, it helps improve insulin resistance, reduce fibrosis, and mitigate inflammation. Nevertheless, high dosages can have adverse effects on the human body [99,100,101]. Cystine is the primary sulphur-containing amino acid and can be considered a semi-essential amino acid because it can be obtained from the diet but is also produced from methionine degradation. This process is important in the human body for stabilising protein structures, such as those found in the hair, skin, and nails. It also plays vital roles in biological processes occurring in the human body, such as lipid biosynthesis, and has an important role in the development of skeletal muscles [102,103].

The levels of phosphorus in the developed pastas varied from 0.07 ± 0.01% db to 0.17 ± 0.01% db. In the work by Torres et al. [69], the phosphorus content of pasta samples varied from 33.9 to 189.0 mg/100 g db (corresponding to 0.03–0.19% db), which are values in a very similar range to those found for the pastas developed in this work. Phosphorus is essential for forming strong bones and teeth, for producing cellular energy, for cell signalling, for brain health, for improved cardiovascular systems, and for homeostasis [104,105,106]. Nevertheless, when in excess, it can have adverse effects, such as cardiovascular and kidney diseases [107,108,109,110,111,112].

Table 10 presents the values obtained for the bioactive compounds analysed in the six pasta samples, revealing significant differences for all the parameters evaluated. Total carotenoids increased with an increasing percentage of carrot powder and were higher in the gluten-free pastas (ranging from 7.23 ± 0.05 to 31.40 ± 0.04 mg/100 g db) as compared with the wheat-based pastas (ranging from 7.73 ± 0.02 to 39.92 ± 0.03 mg/100 g db). Gull et al. [65] reported values of total carotenoids in pasta samples formulated with 4% carrot pomace in the range of 1.3–2.3 µg/100 g dry basis, which is lower compared to our values. Carrot is a natural, rich source of carotenoids, including alpha- and beta-carotene [65,113]. The contents of beta-carotene also increased with increasing carrot powder, being higher for the gluten-free sample with 10% carrot powder (20.19 ± 0.05 mg/100 g db), but lycopene varied in an opposite trend, being higher for the pasta sample containing wheat flour and no added carrot powder (6.81 ± 0.03 mg/100 g db). The concentrations of beta-carotene were higher in the gluten-free samples in comparison to those containing wheat flour, because buckwheat seeds contain various nutrients, such as carbohydrates, in addition to proteins, vitamins, minerals, phytosterols, and phenolic compounds [57]. This corroborates our findings according to which the gluten-free pastas had higher total phenolic contents (232.69 ± 2.54–275.52 ± 4.44 mg GAE/100 g db) compared to the wheat-based pastas (142.45 ± 21.55–155.89 ± 16.48 mg GAE/100 g db). Sujka et al. [57] reported values of total phenolic compounds in pasta samples containing buckwheat hull varying from 120 to 254 mg/100 g db, which increased as the buckwheat percentage increased from 1 to 20%.

It was further observed that the phenolic compound content increased with an increasing percentage of carrot powder. Carrots are a rich source of antioxidant compounds, including phenolic compounds, in addition to carotenes [114,115]. Although it has been reported that cooked pasta contains higher antioxidant activity and total phenolic compounds content compared to uncooked pasta [78], Gull et al. [65] found higher values of total phenolics in uncooked pasta containing carrot pomace (0.67 mg GAE/g) than in cooked samples (0.30 mg GAE/g).

### 3.7. Sensorial Characteristics of the Pasta Samples

**Figure 8:**
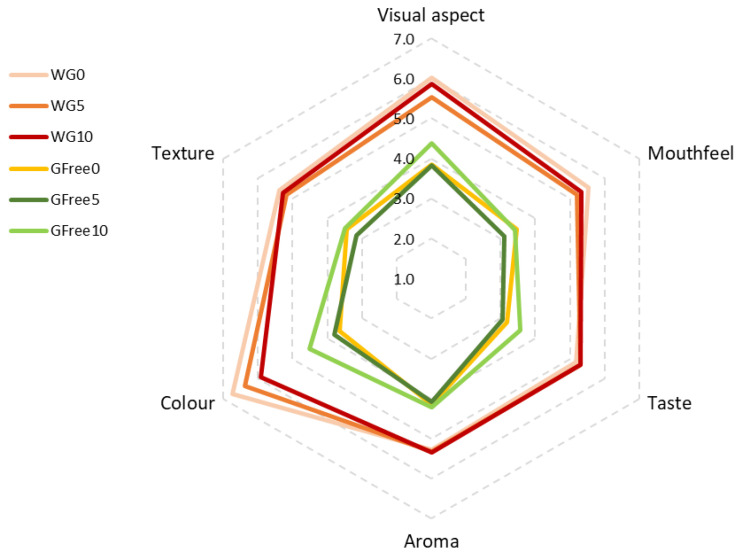
Sensorial descriptors for the six pasta samples. (Scale from 1 = extremely unacceptable to 9 = extremely acceptable).

Figure 8 shows the results obtained from the sensorial analysis of the six pasta samples, calculated as the average scores of all 48 judges. The pasta samples containing gluten were clearly more valued by the panellists than the gluten-free pastas. In fact, the majority of people consume regular pasta made with wheat flour, with those who have celiac disease having to find suitable alternatives.

Table 11 presents the mean values of the scores for each of the attributes analysed in the six pasta samples. Statistical analyses revealed significant differences for all studied sensorial characteristics. The sensorial attributes more valued were colour and visual aspect, particularly in the wheat-based pastas, but also in the buckwheat-based formulations. Attributes such as aroma, taste, or texture were very similar for the three wheat-based pastas, while for the buckwheat pastas, only the attribute of aroma was found to be very similar across the three variations. While the addition of an increased percentage of carrot powder improved the acceptability of colour for the gluten-free pastas, an opposite effect was observed for the pastas containing gluten, for which increasing the addition of carrot powder reduced the acceptability of colour. This might be because the control formulations of both types of pasta were different; the wheat-based one was very clear and turned more orange with the addition of carrot powder, thus moving away from the original colour that people usually associate with pasta. In contrast, the control buckwheat pasta was darker, but adding carrot powder made it lighter and, consequently, more appealing to the panellists.

**Figure 9:**
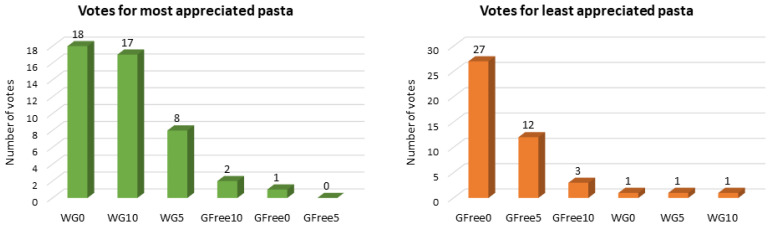
Results for the most and least appreciated pasta samples.

Figure 9 illustrates the results for questions regarding the most and least preferred samples. The results clearly show that the samples containing wheat flour were preferred, considering the overall appreciation of the sensorial characteristics, especially the control (WG0), which was preferred by 18 judges, and the sample with the highest percentage of carrot powder (WG10), which was preferred by 17 judges. On the other hand, the least appreciated sample was the buckwheat control pasta (GFree0) (*n* = 27), which aligns with the previously discussed sensorial descriptors of the pasta samples.

## 4. Conclusions

The results allowed us to conclude that the hydration and cooking characteristics of the wheat-based pasta samples were superior to those of the gluten-free buckwheat-based pasta samples, which presented higher water uptake and cooking yield at the cost of integrity. Textual properties indicated that the gluten-free pasta samples were significantly less cohesive, exhibiting lower springiness and resilience. For the pastas containing wheat flour, the hardness was higher for the sample with the highest carrot powder content, while an opposite trend was observed for the buckwheat pasta. Sensorial evaluation confirmed that the texture was better rated for pastas containing wheat flour than for those containing buckwheat flour.

Pasting properties showed a higher pasting temperature and peak viscosity for the gluten-free samples. However, while the pasting temperature increased with the addition of carrot powder, the peak viscosity did not exhibit the same trend for both types of pasta.

The gluten-free buckwheat-based pasta samples were darker, but lightness increased with the addition of carrot powder, and so did redness and yellowness. Still, the colour of the wheat-based pasta samples was lighter and had a more intense redness and yellowness. The sensory evaluation confirmed that the judges preferred the wheat-based pasta samples in terms of colour acceptability.

Chemical evaluations confirmed that the buckwheat pasta samples were richer in comparison to the gluten-based pastas in terms of protein, ash, and fat, while containing less carbohydrates and starch. Additionally, the gluten-free pastas contained higher amounts of amino acids, phosphorus, carotenoids, and phenolic compounds. However, the scores of the sensory panel members were lower for the visual aspect, aroma, taste, and mouthfeel of the gluten-free samples.

As for the addition of carrot powder, it was confirmed that an increasing percentage of carrot powder allowed us to obtain pastas with higher total carotenoids, beta-carotene, and total phenolic compounds. While the panellists did not attribute higher scores to wheat-based pasta samples with an increasing percentage of carrot powder, they showed an opposite trend for the wheat-based pastas, attributing higher scores to the samples with a higher percentage of carrot powder for the properties of visual aspect, taste, aroma, colour, and texture.

Finally, the panellists preferred the control wheat-based pasta and that with 10% carrot powder. For the buckwheat-based pastas, the preferred sample was also the one containing 10% carrot powder.

## Figures and Tables

**Table 1:** Formulations used to prepare the pasta samples.

Formulations								
Sample Code ^1^	Dehydrated Carrot %	Wheat Flour (g)	Buckwheat Four (g)	Egg (g)	Olive Oil (g)	Carrot Powder (g)	Total Dough After Kneading (g)	Total Pasta After Moulding the Strips (g)
WG0	0%	100.47	0.00	55.11	5.68	0.00	157.48	154.59
WG5	5%	95.47	0.00	57.03	5.12	5.03	156.32	155.66
WG10	10%	90.80	0.00	53.07	5.39	10.02	158.27	156.35
GFree0	0%	0.00	100.10	56.69	5.76	0.00	148.28	144.71
GFree5	5%	0.00	95.17	44.43	5.09	4.95	146.39	143.15
GFree10	10%	0.00	90.03	73.78	5.01	10.12	160.11	148.43

^1^ WG = with gluten, GFree = gluten free.

**Table 2:** Programs used to perform the rheological measurements with RVA.

Wheat Pasta Samples			Gluten-Free Pasta Samples (with Buckwheat)		
Time (hh:mm:ss)	Function (Unit)	Value	Time (hh:mm:ss)	Function (Unit)	Value
00:00:00	Temp (°C)	50	00:00:00	Temp (°C)	25
00:00:00	Speed (rpm)	960	00:00:00	Speed (rpm)	960
00:00:10	Speed (rpm)	160	00:00:10	Speed (rpm)	160
00:01:00	Temp (°C)	50	00:01:00	Temp (°C)	25
00:05:00	Temp (°C)	95	00:04:45	Temp (°C)	95
00:07:30	Temp (°C)	95	00:07:15	Temp (°C)	95
00:11:30	Temp (°C)	50	00:11:00	Temp (°C)	25
00:13:00	Temp (°C)	50	00:11:30	END	
00:13:30	END				

**Table 3:** Drying of the pasta samples.

Sample Code ^1^	Carrot Powder %	Weight Before Drying (g)	Drying Temperature (°C)	Drying Time (hh:mm)	Weight After Drying (g)	Drying Loss (%)	Final Moisture Content Mean ± SD (%) ^2^
WG0	0%	77.40	60	04:05	59.01	23.76	0.72 ± 0.19
WG5	5%	78.50	60	04:15	59.10	24.71	0.55 ± 0.22
WG10	10%	77.55	60	04:05	59.03	23.88	0.60 ± 0.31
GFree0	0%	72.37	60	04:15	51.76	28.48	1.15 ± 0.23
GFree5	5%	71.41	60	04:10	51.30	28.16	1.48 ± 0.10
GFree10	10%	74.15	60	04:00	53.88	27.34	0.81 ± 0.13

^1^ WG = with gluten, GFree = gluten free. ^2^ Mean and standard deviation calculated from three measurements.

**Table 4:** Cooking properties of the pasta samples.

Sample Code ^1^	Carrot Powder (%)	State	Optimal Cooking Time (mm:ss)	Weight Before Cooking (g)	Weight After Cooking (g)	Weight of Residue Lost During Cooking (g)	Cooking Yield (%)	Cooking Loss (%)	Water Absorption (%)
WG0	0%	Fresh	10:00	20.81	36.79	0.82	177	3.92	77
WG5	5%	Fresh	09:30	20.06	35.28	1.02	176	5.08	76
WG10	10%	Fresh	09:00	20.09	34.26	0.94	171	4.69	71
GFree0	0%	Fresh	08:00	20.38	37.49	0.49	184	2.42	84
GFree5	5%	Fresh	07:30	20.30	39.95	0.82	197	4.06	97
GFree10	10%	Fresh	07:30	20.03	37.5	0.99	187	4.95	87
WG0	0%	Dried	08:00	20.22	51.82	0.88	240	4.37	140
WG5	5%	Dried	08:00	20.06	51.82	1.10	258	5.51	158
WG10	10%	Dried	07:30	20.02	48.78	1.26	244	6.31	144
GFree0	0%	Dried	07:00	20.20	58.30	0.71	289	3.53	189
GFree5	5%	Dried	06:30	20.06	49.56	0.95	247	4.73	147
GFree10	10%	Dried	06:30	20.06	45.81	0.78	228	3.89	128

^1^ WG = with gluten, GFree = gluten free.

**Table 5:** Biometric characteristics of the different pasta samples.

Sample Code ^1^	Carrot Powder (%)	State	Width ^2,3^	Thickness ^2,3^
WG0	0%	Fresh	6.08 ± 0.24 ^abA^	2.10 ± 0.18 ^bA^
WG5	5%	Fresh	5.83 ± 0.27 ^aA^	2.25 ± 0.14 ^bA^
WG10	10%	Fresh	5.80 ± 0.14 ^aA^	2.13 ± 0.15 ^bAB^
GFree0	0%	Fresh	6.57 ± 0.18 ^cB^	1.45 ± 0.19 ^aA^
GFree5	5%	Fresh	6.43 ± 0.16 ^bcA^	1.37 ± 0.08 ^aA^
GFree10	10%	Fresh	5.80 ± 0.28 ^aA^	2.07 ± 0.19 ^bA^
WG0	0%	Fresh-cooked	7.51 ± 0.56 ^bcdB^	2.48 ± 0.48 ^bAB^
WG5	5%	Fresh-cooked	7.82 ± 0.46 ^cdB^	2.77 ± 0.27 ^bB^
WG10	10%	Fresh-cooked	8.00 ± 0.26 ^dC^	2.67 ± 0.12 ^bB^
GFree0	0%	Fresh-cooked	6.62 ± 0.33 ^aB^	1.53 ± 0.21 ^aA^
GFree5	5%	Fresh-cooked	7.20 ± 0.35 ^abcB^	1.48 ± 0.19 ^aA^
GFree10	10%	Fresh-cooked	7.02 ± 0.44 ^abB^	1.95 ± 0.16 ^aA^
WG0	0%	Dried	6.45 ± 0.25 ^aA^	2.63 ± 0.32 ^dB^
WG5	5%	Dried	6.10 ± 0.30 ^aA^	2.25 ± 0.15 ^cdA^
WG10	10%	Dried	6.03 ± 0.19 ^aA^	2.03 ± 0.15 ^bcA^
GFree0	0%	Dried	6.18 ± 0.17 ^aA^	1.65 ± 0.11 ^abAB^
GFree5	5%	Dried	6.15 ± 0.08 ^aA^	1.42 ± 0.10 ^aA^
GFree10	10%	Dried	6.03 ± 0.58 ^aA^	2.18 ± 0.39 ^cA^
WG0	0%	Dried-cooked	7.53 ± 0.42 ^dB^	2.93 ± 0.23 ^cB^
WG5	5%	Dried-cooked	5.60 ± 0.55 ^aA^	2.52 ± 0.24 ^bcAB^
WG10	10%	Dried-cooked	6.45 ± 0.20 ^bB^	2.42 ± 0.36 ^bBC^
GFree0	0%	Dried-cooked	7.45 ± 0.19 ^cdC^	1.85 ± 0.14 ^aB^
GFree5	5%	Dried-cooked	6.95 ± 0.19 ^bcB^	1.75 ± 0.15 ^aB^
GFree10	10%	Dried-cooked	7.53 ± 0.19 ^dB^	2.10 ± 0.28 ^abA^

^1^ WG = with gluten, GFree = gluten free. ^2^ Mean values in the same column with different superscript small letters correspond to significant differences when comparing between pasta samples for a given state (ANOVA with Tukey test, *p* < 0.05). ^3^ Mean values in the same column with different superscript capital letters correspond to significant differences when comparing between states for a given pasta sample (ANOVA with Tukey test, *p* < 0.05).

**Table 6:** Colour coordinates of the different pasta samples.

Sample Code ^1^	Carrot Powder (%)	State	L* ^2,3^	a* ^2,3^	b* ^2,3^	TCD ^4^
WG0 (Control Fresh)	0%	Fresh	68.60 ± 0.57 ^cBC^	3.84 ± 0.04 ^aD^	40.62 ± 4.62 ^bcB^	—
WG5	5%	Fresh	67.39 ± 3.98 ^cAB^	10.55 ± 2.59 ^cC^	44.07 ± 6.18 ^cB^	7.64
WG10	10%	Fresh	67.25 ± 1.09 ^cB^	14.95 ± 0.69 ^dC^	51.24 ± 2.70 ^dB^	15.43
GFree0	0%	Fresh	55.81 ± 1.12 ^bC^	7.85 ± 0.33 ^bC^	21.01 ± 1.83 ^aC^	23.76
GFree5	5%	Fresh	48.25 ± 5.08 ^aB^	7.96 ± 1.18 ^bC^	27.51 ± 3.27 ^aB^	24.56
GFree10	10%	Fresh	46.06 ± 4.91 ^aB^	10.94 ± 1.95 ^cC^	35.38 ± 3.73 ^bB^	24.21
WG0 (Control Fresh-cooked)	0%	Fresh-cooked	71.84 ± 1.49 ^dC^	0.41 ± 0.18 ^aB^	19.12 ± 2.36 ^bA^	—
WG5	5%	Fresh-cooked	59.20 ± 8.85 ^cA^	1.74 ± 0.88 ^abA^	27.86 ± 4.73 ^cA^	15.43
WG10	10%	Fresh-cooked	66.48 ± 2.34 ^cdB^	3.03 ± 1.79 ^bcA^	30.17 ± 7.55 ^cA^	12.55
GFree0	0%	Fresh-cooked	48.59 ± 4.53 ^bB^	5.61 ± 0.31 ^dB^	6.03 ± 1.20 ^aA^	27.18
GFree5	5%	Fresh-cooked	31.37 ± 5.93 ^aA^	3.95 ± 1.24 ^cdA^	14.68 ± 2.61 ^bA^	40.87
GFree10	10%	Fresh-cooked	30.31 ± 7.06 ^aA^	3.43 ± 0.68 ^bcA^	16.05 ± 3.39 ^bA^	41.75
WG0 (Control Dried)	0%	Dried	35.87 ± 4.58 ^aA^	2.91 ± 0.25 ^aC^	16.33 ± 1.73 ^aA^	—
WG5	5%	Dried	68.95 ± 2.49 ^dB^	6.38 ± 1.18 ^cB^	42.72 ± 2.50 ^cB^	42.46
WG10	10%	Dried	62.38 ± 4.25 ^cdAB^	6.91 ± 0.51 ^cB^	47.19 ± 1.46 ^dB^	40.88
GFree0	0%	Dried	46.08 ± 1.56 ^bB^	4.54 ± 0.56 ^bA^	15.06 ± 1.35 ^aB^	10.41
GFree5	5%	Dried	56.61 ± 2.12 ^cC^	5.79 ± 0.59 ^bcB^	30.78 ± 1.73 ^bB^	25.44
GFree10	10%	Dried	32.11 ± 8.16 ^aA^	5.62 ± 1.20 ^bcB^	32.08 ± 3.51 ^bB^	16.42
WG0 (Control Dried-cooked)	0%	Dried-cooked	67.27 ± 2.91 ^dB^	−0.11 ± 0.09 ^aA^	18.32 ± 1.17 ^bA^	—
WG5	5%	Dried-cooked	63.50 ± 4.79 ^cdAB^	1.55 ± 1.02 ^bA^	34.54 ± 4.33 ^cA^	16.73
WG10	10%	Dried-cooked	58.54 ± 3.64 ^cA^	2.49 ± 1.26 ^bcA^	35.45 ± 2.02 ^cA^	19.40
GFree0	0%	Dried-cooked	34.62 ± 4.96 ^bA^	4.32 ± 0.53 ^dA^	5.87 ± 0.68 ^aA^	35.22
GFree5	5%	Dried-cooked	26.64 ± 2.16 ^cdA^	3.74 ± 0.54 ^cdA^	15.81 ± 1.79 ^bA^	40.89
GFree10	10%	Dried-cooked	33.60 ± 4.17 ^cdA^	3.05 ± 0.36 ^cdA^	18.20 ± 1.83 ^bA^	33.82

^1^ WG = with gluten, GFree = gluten free. ^2^ Mean values in the same column with different superscript small letters correspond to significant differences when comparing between pasta samples for a given state (ANOVA with Tukey test, *p* < 0.05). ^3^ Mean values in the same column with different superscript capital letters correspond to significant differences when comparing between states for a given pasta sample (ANOVA with Tukey test, *p* < 0.05). ^4^ TCD = total colour difference (calculated from the mean values of each sample and the control sample).

**Table 7:** Rheological properties of the different dried uncooked pasta samples.

Sample Code ^1^	Carrot Powder (%)	Pasting Temperature (°C)	Peak Time (mm:ss)	Peak Viscosity (Pa.s)	Breakdown Viscosity (Pa.s)	Final Viscosity (Pa.s)	Setback Viscosity (Pa.s)
WG0	0%	67.65 ± 1.41	04:56 ± 00:11	0.761 ± 0.037	0.136 ± 0.016	1.818 ± 0.132	1.193 ± 0.078
WG5	5%	73.93 ± 9.10	05:12 ± 00:00	1.430 ± 0.264	0.387 ± 0.183	2.302 ± 0.074	1.260 ± 0.013
WG10	10%	84.43 ± 1.10	05:20 ± 00:05	1.576 ± 0.128	0.714 ± 0.081	1.812 ± 0.105	0.950 ± 0.059
GFree0	0%	87.17 ± 0.58	07:59 ± 00:00	1.832 ± 0.095	0.251 ± 0.050	4.808 ± 0.284	3.226 ± 0.145
GFree5	5%	87.93 ± 0.63	07:00 ± 00:00	1.904 ± 0.070	0.187 ± 0.014	5.560 ± 0.214	3.844 ± 0.141
GFree10	10%	88.35 ± 0.07	06:21 ± 00:06	1.730 ± 0.018	0.166 ± 0.008	4.524 ± 0.035	2.959 ± 0.008

^1^ WG = with gluten, GFree = gluten free.

**Table 8:** Textural parameters of the different pasta samples.

Sample Code ^1^	Carrot Powder (%)	State	Hardness ^2,3^ (N)	Resilience ^2,3^ (%)	Cohesiveness ^2,3^ (%)	Springiness ^2,3^ (%)	Chewiness ^2,3^ (N)
WG0	0%	Fresh	6.91 ± 2.32 ^aA^	7.86 ± 0.28 ^aA^	36.15 ± 2.09 ^cA^	44.79 ± 2.21 ^dA^	1.13 ± 0.41 ^aA^
WG5	5%	Fresh	13.68 ± 0.50 ^aA^	9.63 ± 1.72 ^aA^	28.62 ± 2.12 ^bA^	40.58 ± 2.18 ^dA^	1.57 ± 0.22 ^aA^
WG10	10%	Fresh	65.91 ± 5.89 ^cA^	38.98 ± 1.02 ^cA^	43.73 ± 1.15 ^dA^	34.83 ± 3.30 ^cA^	10.13 ± 2.09 ^dA^
GFree0	0%	Fresh	32.66 ± 5.14 ^bB^	9.96 ± 0.59 ^aA^	20.55 ± 0.92 ^aA^	24.96 ± 1.13 ^abA^	1.61 ± 0.30 ^abA^
GFree5	5%	Fresh	66.10 ± 11.92 ^cA^	17.99 ± 1.80 ^bA^	22.98 ± 2.04 ^aA^	27.38 ± 5.33 ^bA^	3.63 ± 0.73 ^bA^
GFree10	10%	Fresh	68.35 ± 4.89 ^cB^	37.54 ± 2.50 ^cA^	35.02 ± 3.11 ^cA^	21.00 ± 3.58 ^aA^	5.82 ± 0.52 ^cAB^
WG0	0%	Fresh-cooked	7.42 ± 0.54 ^aA^	44.56 ± 2.76 ^aA^	61.83 ± 14.64 ^abB^	96.00 ± 1.94 ^cB^	4.42 ± 1.19 ^aA^
WG5	5%	Fresh-cooked	11.12 ± 3.78 ^abA^	50.77 ± 20.91 ^abB^	57.26 ± 22.78 ^abB^	73.45 ± 15.78 ^abB^	6.89 ± 3.30 ^abA^
WG10	10%	Fresh-cooked	23.53 ± 6.00 ^cA^	73.31 ± 8.10 ^bB^	65.37 ± 2.96 ^bBC^	70.00 ± 10.55 ^aB^	10.53 ± 2.17 ^bA^
GFree0	0%	Fresh-cooked	15.95 ± 2.59 ^abcAB^	32.89 ± 5.89 ^aB^	50.04 ± 14.82 ^abB^	90.20 ± 3.75 ^bcD^	7.46 ± 3.26 ^abA^
GFree5	5%	Fresh-cooked	13.98 ± 5.60 ^abcA^	37.28 ± 8.09 ^aB^	40.87 ± 6.75 ^aB^	70.08 ± 13.63 ^aB^	4.08 ± 1.38 ^aA^
GFree10	10%	Fresh-cooked	20.04 ± 6.83 ^bcA^	71.19 ± 15.54 ^abB^	60.01 ± 5.73 ^abB^	69.50 ± 5.52 ^aC^	8.20 ± 2.37 ^abB^
WG0	0%	Dried	213.79 ± 38.32 ^bB^	83.58 ± 13.65 ^abcB^	57.37 ± 18.03 ^aB^	63.67 ± 10.45 ^bB^	75.27 ± 19.43 ^abB^
WG5	5%	Dried	183.72 ± 47.57 ^aB^	72.68 ± 10.81 ^aB^	70.93 ± 11.25 ^abB^	61.83 ± 12.90 ^bB^	99.35 ± 25.59 ^abB^
WG10	10%	Dried	182.67 ± 15.40 ^abB^	91.08 ± 3.30 ^bcC^	71.87 ± 18.90 ^abC^	58.88 ± 16.18 ^abB^	75.38 ± 17.38 ^abB^
GFree0	0%	Dried	252.29 ± 17.12 ^bC^	91.32 ± 3.97 ^bcB^	86.36 ± 1.64 ^bC^	71.75 ± 5.24 ^bC^	105.32 ± 71.44 ^abB^
GFree5	5%	Dried	221.45 ± 14.62 ^bB^	98.73 ± 4.39 ^cB^	88.02 ± 5.10 ^bC^	65.75 ± 18.37 ^bB^	138.19 ± 13.05 ^bB^
GFree10	10%	Dried	95.93 ± 19.12 ^aC^	75.65 ± 9.16 ^abB^	70.30 ± 3.99 ^abB^	35.42 ± 6.00 ^aB^	25.30 ± 7.12 ^aC^
WG0	0%	Dried-cooked	5.16 ± 2.02 ^abA^	46.01 ± 18.19 ^aA^	52.07 ± 3.58 ^bAB^	60.50 ± 6.05 ^cB^	1.65 ± 0.72 ^aA^
WG5	5%	Dried-cooked	4.50 ± 1.94 ^aA^	45.93 ± 20.49 ^aB^	53.78 ± 7.51 ^bB^	62.17 ± 9.72 ^cB^	1.62 ± 0.99 ^aA^
WG10	10%	Dried-cooked	14.18 ± 3.57 ^bA^	73.55 ± 5.29 ^bB^	53.79 ± 4.44 ^bAB^	58.08 ± 5.01 ^bcB^	4.46 ± 1.25 ^bA^
GFree0	0%	Dried-cooked	13.37 ± 9.55 ^abA^	33.83 ± 12.41 ^aB^	28.17 ± 5.19 ^aA^	44.30 ± 11.15 ^abB^	1.91 ± 1.81 ^aA^
GFree5	5%	Dried-cooked	12.70 ± 6.80 ^abA^	28.63 ± 8.47 ^aB^	27.77 ± 2.37 ^aA^	43.33 ± 4.90 ^abA^	1.46 ± 0.65 ^aA^
GFree10	10%	Dried-cooked	5.64 ± 3.14 ^abA^	28.06 ± 13.91 ^aA^	33.77 ± 12.01 ^aA^	41.33 ± 12.89 ^aB^	0.95 ± 0.76 ^aA^

^1^ WG = with gluten, GFree = gluten free. ^2^ Mean values in the same column with different superscript small letters correspond to significant differences when comparing between pasta samples for a given state (ANOVA with Tukey test, *p* < 0.05). ^3^ Mean values in the same column with different superscript capital letters correspond to significant differences when comparing between states for a given pasta sample (ANOVA with Tukey test, *p* < 0.05).

**Table 9:** Chemical properties of the six pasta samples.

Measurements	WG0 ^3^	WG5 ^3^	WG10 ^3^	GFree0 ^3^	GFree5 ^3^	GFree10 ^3^
Moisture (% wb) ^1^	62.95 ± 1.43 ^abc^	67.90 ± 1.05 ^c^	58.55 ± 2.40 ^a^	62.06 ± 1.64 ^ab^	64.52 ± 1.69 ^bc^	64.92 ± 3.28 ^bc^
Fat (% db) ^2^	12.91 ± 0.00 ^e^	11.61 ± 0.14 ^b^	11.11 ± 0.12 ^a^	12.04 ± 0.05 ^c^	12.53 ± 0.02 ^d^	13.30 ± 0.07 ^f^
Protein (% db) ^2^	15.06 ± 0.02 ^a^	15.47 ± 0.01 ^b^	14.99 ± 0.18 ^a^	18.34 ± 0.11 ^d^	17.03 ± 0.09 ^c^	18.38 ± 0.21 ^d^
Ash (% db) ^2^	1.41 ± 0.01 ^a^	1.76 ± 0.01 ^b^	1.69 ± 0.03 ^b^	1.71 ± 0.03 ^b^	1.74 ± 0.02 ^b^	2.45 ± 0.11 ^c^
Carbohydrates (% db) ^2^	70.61 ± 0.10 ^d^	71.16 ± 0.15 ^e^	72.21 ± 0.13 ^f^	67.92 ± 0.14 ^b^	68.70 ± 0.11 ^c^	65.87 ± 0.28 ^a^
Starch (% db) ^2^	64.08 ± 0.24 ^c^	62.67 ± 0.17 ^c^	62.65 ± 0.98 ^c^	59.18 ± 0.49 ^b^	58.35 ± 0.71 ^b^	55.70 ± 0.62 ^a^
Lysine (% db) ^2^	1.44 ± 0.03 ^b^	1.45 ± 0.01 ^b^	1.30 ± 0.06 ^a^	1.55 ± 0.01 ^c^	1.45 ± 0.01 ^b^	1.60 ± 0.02 ^c^
Cysteine (% db) ^2^	0.48 ± 0.0 ^b^	0.56 ± 0.01 ^c^	0.45 ± 0.01 ^a^	0.54 ± 0.00 ^c^	0.50 ± 0.01 ^b^	0.65 ± 0.01 ^d^
Methionine (% db) ^2^	0.23 ± 0.00 ^a^	0.24 ± 0.00 ^b^	0.24 ± 0.00 ^bc^	0.24 ± 0.00 ^bc^	0.27 ± 0.00 ^d^	0.25 ± 0.00 ^c^
Phosphorus (% db) ^2^	0.10 ± 0.01 ^a^	0.07 ± 0.01 ^a^	0.13 ± 0.01 ^b^	0.17 ± 0.01 ^c^	0.13 ± 0.01 ^b^	0.10 ± 0.01 ^a^

^1^ Values are in percentage wet basis (wb) and correspond to mean and standard deviation from three measurements. ^2^ Values converted to dry basis (db) and correspond to mean and standard deviation from three measurements. ^3^ Mean values in the same line with different superscript letters correspond to significant differences between pasta samples (ANOVA with Tukey test, *p* < 0.05).

**Table 10:** Bioactive compounds (carotenoids and phenolic compounds) of the six pasta samples.

Measurements (mg/100 g db) ^1^	WG0 ^3^	WG5 ^3^	WG10 ^3^	GFree0 ^3^	GFree5 ^3^	GFree10 ^3^
Total carotenoids	7.23 ± 0.05 ^a^	12.70 ± 0.04 ^c^	31.40 ± 0.04 ^e^	7.73 ± 0.02 ^b^	17.25 ± 0.02 ^d^	39.92 ± 0.03 ^f^
Beta-carotene	0.02 ± 0.00 ^a^	5.69 ± 0.07 ^c^	16.84 ± 0.06 ^e^	2.45 ± 0.04 ^b^	6.89 ± 0.09 ^d^	20.19 ± 0.05 ^f^
Lycopene	6.81 ± 0.03 ^f^	2.96 ± 0.01 ^c^	0.91 ± 0.02 ^a^	4.73 ± 0.06 ^e^	3.64 ± 0.04 ^d^	2.13 ± 0.02 ^b^
Total phenolic compounds ^2^	142.45 ± 21.55 ^a^	145.03 ± 9.59 ^a^	155.89 ± 16.48 ^a^	232.69 ± 2.54 ^b^	241.36 ± 17.75 ^b^	275.52 ± 4.44 ^b^

^1^ Values expressed in dry basis (db) and correspond to mean and standard deviation from three measurements. ^2^ Expressed in GAE—Gallic Acid Equivalents. ^3^ Mean values in the same line with different superscript letters correspond to significant differences between pasta samples (ANOVA with Tukey test, *p* < 0.05).

**Table 11:** Mean scores for the sensorial attributes analysed in the six pasta samples.

Sensorial Attributes ^1^	WG0 ^2^	WG5 ^2^	WG10 ^2^	GFree0 ^2^	GFree5 ^2^	GFree10 ^2^
Visual aspect	6.0 ± 1.6 ^b^	5.5 ± 1.7 ^b^	5.9 ± 1.8 ^b^	3.9 ± 2.2 ^a^	3.8 ± 2.0 ^a^	4.4 ± 2.0 ^a^
Mouthfeel	5.5 ± 2.0 ^b^	5.2 ± 1.6 ^b^	5.3 ± 1.9 ^b^	3.5 ± 1.8 ^a^	3.1 ± 1.7 ^a^	3.4 ± 1.9 ^a^
Taste	5.2 ± 2.0 ^b^	5.3 ± 1.9 ^b^	5.3 ± 2.0 ^b^	3.2 ± 1.6 ^a^	3.1 ± 1.7 ^a^	3.6 ± 1.9 ^a^
Aroma	5.3 ± 2.4 ^ab^	5.3 ± 2.1 ^ab^	5.4 ± 2.0 ^b^	4.2 ± 2.1 ^ab^	4.1 ± 2.1 ^a^	4.2 ± 2.0 ^ab^
Colour	6.8 ± 1.7 ^b^	6.4 ± 2.0 ^b^	5.9 ± 2.3 ^b^	3.6 ± 2.3 ^a^	3.8 ± 2.1 ^a^	4.5 ± 2.1 ^a^
Texture	5.4 ± 2.3 ^b^	5.2 ± 1.9 ^b^	5.3 ± 2.0 ^b^	3.4 ± 2.0 ^a^	3.1 ± 2.0 ^a^	3.5 ± 1.9 ^a^

^1^ Scale from 1 = extremely unacceptable to 9 = extremely acceptable. ^2^ Mean values in the same line with different superscript letters correspond to significant differences between pasta samples (ANOVA with Tukey test, *p* < 0.05).

## Data Availability

The original contributions presented in this study are included in the article. Further inquiries can be directed to the corresponding author.
